# Adjuvant radiochemotherapy in locally advanced gastric cancer: from evidence to daily clinical practice in a single institution

**DOI:** 10.3332/ecancer.2020.1137

**Published:** 2020-11-05

**Authors:** Manuel González-Domingo, Cristóbal Ulloa, Jorge Olivares, Sebastián Estrada, Pablo González, Neyla Cardozo

**Affiliations:** 1 Department of Radiation Oncology, Instituto Oncológico, Viña del Mar, 2540364, Chile; 2 Surgery resident, University of Valparaíso, Chile; 3 Oncology and Radiotherapy resident, University of Valparaíso, Chile; 4 Department of Radiation Oncology, Arturo López Pérez Foundation, Santiago, Chile

**Keywords:** gastric cancer, radiotherapy, overall survival, radiochemotherapy, clinical practice

## Abstract

**Background:**

Gastric cancer is one of the main important causes of cancer death in Chile.

**Objective:**

To report the results of adjuvant radiochemotherapy in advanced gastric cancer.

**Material and Methods:**

Between 2000 and 2018, 214 subjects aged 23–85 (median, 62) years with lymph node and/or serosa involvement were treated with adjuvant chemoradiotherapy after curative resection.

**Results:**

With a median follow-up of 41 months, overall 3- and 5-year survival was 54.9% and 40.85%, respectively. On multivariate analysis, the factors associated with lower survival were aged >65 years, stage group and number of lymph nodes involved.

**Conclusion:**

In patients with locoregionally advanced gastric cancer treated with curative intent with surgery and adjuvant radiochemotherapy, the overall 5-year survival reported from local clinical practice is similar to that reported in randomised series and supports its use as an effective treatment for this type of patients in our country.

## Introduction

The incidence of gastric cancer worldwide for 2018 was 15.7 per 100,000 inhabitants [1].

Gastric cancer ranks third after lung and liver cancers for low- and middle-income countries. Chile is among the countries with the highest incidence rates, along with Japan, Costa Rica and Singapore [1].

When analysing Chile’s national epidemiology, the incidence of gastric cancer in men is 29.7 per 100,000 inhabitants, being second only to prostate cancer. On the contrary, it is in the fifth place for women, with an incidence rate of 14.3 per 100,000 inhabitants, after breast, non-melanoma skin, gallbladder, biliary tract and cervix cancers [2].

When analysing the specific causes of mortality by the type of cancer and sex in 2015, we found that the main causes of death in men were gastric cancer, with 2,247 deaths (25.2 per 100,000 inhabitants), followed by prostate cancer, while gastric cancer was in the fourth place for women, with a rate of 12.3 per 100,000 inhabitants after breast and lung cancers [3].

In the Valparaíso Region, the adjusted regional mortality rate exceeds the national one for men, with 29.8 per 100,000 inhabitants [3].

Radical (R0) surgical resection is the standard surgical treatment for gastric cancer. Subtotal or total gastrectomy with D2 lymph node dissection is recommended in patients with regionally advanced gastric cancer [5]. However, for stage-II and -III diseases [4], surgery alone is not sufficient for cure.

The 5-year survival rate decreases rapidly with the increase of the disease stage. Among western patients with localised lesions, the 5-year survival rate is 65%; however, for those with regional spread, the estimated 5-year survival is reduced to 30% (6.7). This poor prognosis among resected patients promoted the development of several studies that supported adjuvant strategies.

The purpose of our study is to evaluate the results of adjuvant radiochemotherapy for gastric cancer patients undergoing surgery with curative intent in clinical practice at our institution and to compare their results with those published to date.

## Materials and methods

The study presents a retrospective cohort of all patients diagnosed with locally advanced gastric cancer (T1-4, N1-3, M0) who received surgery plus fluoropyrimidine-based adjuvant radiochemotherapy with curative intent between 2000 and 2018 at the Viña del Mar Cancer Institute; follow up was adjourned to March 2020. All patients underwent surgery at different centres in the V region and were referred to the Cancer Institute. Two-hundred and 14 patients received adjuvant radiochemotherapy, of whom 60 (28%) were women and 154 (72%) men. The median age was 62 years (range 85–23 years). In 12 patients (5.6%), the tumour invaded to the submucosa; in 24 (11%), it reached the muscularis propria; in 44 (20.5%), it penetrated the subserosa without invasion of the visceral peritoneum and in 134 (63%), the tumour invaded the serosa. Eighty-five per cent of the patients had lymph node involvement and one-third of them had seven or more involved nodes. T and N stage distribution and disease stages are presented in Table 1. The average number of lymph nodes examined was 22. Of the 214 patients, 60.7% had >16 lymph nodes studied and only in 29 patients were <10 lymph nodes studied.

All patients were treated with a three-dimensional conformational technique by a high-energy dual-linear accelerator (6–10 MV), receiving a dose of 45 Gy in 25 fractions on the bed, regional nodes and anastomosis or a total-abdomen radiotherapy (WAI) regimen, according to the GOCCHI RATONES study [8]. Chemotherapy was fluoropyrimidine-based, and the most used regimen was daily oral capecitabine. Patients’ characteristics are presented in Table 2.

All patients were staged according to the classification of the American Joint Committee on Cancer (AJCC) 8th Edition [4].

Survival analysis was performed using the Kaplan–Meier method, and survival data were obtained from the Chilean Civil Registry. The log-rank method was used for comparisons of survival between groups. All analyses were performed by STATA 16.

## Results

**Figure 1.**
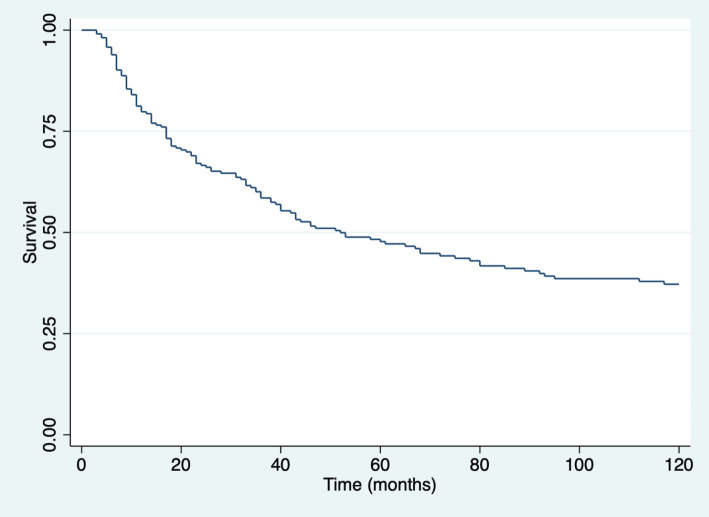
Overall survival (time in months) for all patients.

With a median follow-up of 41 months for the entire group (123 months for living patients), the overall survival at 3 and 5 years for the entire group was 54.9% and 40.85%, respectively (Figure 1). Overall 5-year survival according to sex was 46% in females and 37% in males (*p* = 0.08).

In univariate analysis, of the factors explored, those with a poor prognosis that significantly affected survival were the number of lymph nodes involved (*p* = 0.000), aged ≥ 65 years (*p* = 0.001) and tumour stage (*p* = 0.022). Overall 5-year survival according to nodal stage was 61.2% in N0, 54.5% in pN1, 37.3% in pN2, 26% in pN3a and 28% in pN3b patients (*p* = 0.00). Overall 5-year survival was 50% for patients aged <65 years and 29.9% for those aged ≥65 years. Overall 5-year survival according to stage was 58% for pT1, 45.8% for pT2, 50% for pT3 and 35.3% for pT4a.

In multivariate analysis, the factor with a poor prognosis for overall survival was the number of lymph nodes involved, with a HR of 1.40 for each increased N stage (*p* = 0.00), with a HR of 1.78 for those aged 65 years (*p* = 0.00) and a HR of 1.35 for those with more advanced tumour stages (*p* = 0.01) (Table 3).

## Discussion

Extended D2 surgery in gastric cancer achieves survival rates of less than 50% at 5 years [9]. In the best series, survival is around 30%–40% when there are advanced locoregional compromise factors [10].

Regarding the national literature, there are some data published with exclusive surgery, with similar results [18, 19].

In 2001, the pivotal US Intergroup trial 01164 first reported the results of postoperative radiochemotherapy versus surgery alone establishing postoperative radiochemotherapy in addition to surgery as the standard treatment for gastric cancer. This regimen was shown to improve both locoregional control and overall survival compared to surgery alone in medically fit patients who had microscopically radical resection [6]. A subsequent publication with a 10-year follow-up confirmed the results of the first publication, with an absolute benefit in overall survival (OS) of 9%, with a 1.32 hazard ratio (HR) (95% CI: 1.1–1.6; *p* = 0.0046). Relapse-free survival (RFS) was 11% with an HR of 1.51 (95% CI: 1.25–1.83; *p* < 0.001) [11]. No late toxicity was reported during the long-term follow-up.

Regarding perioperative chemotherapy alone, without the addition of Radiotherapy, in 2006, Cunningham *et al* [12] showed that chemotherapy with epirubicin, cisplatin and 5-FU for six cycles, three administered pre-operatively and three post-operatively, was associated with tumour shrinkage, tumour downstaging and significant overall-survival benefit compared to surgery alone. With a median follow-up of 4 years, the perioperative chemotherapy group had a higher probability of overall survival (HR: 0.75, 95% CI, 0.60–0.93, *p* = 0.009) and a better survival rate at five years, 36% compared to 23% in the surgery group. Thirty-six patients in the perioperative chemotherapy group (14.4%) presented local recurrence versus 52 patients (20.6%) in the surgery group, indicating a lack of locoregional control. Finally, the protocol could only be completed by 42% of the patients assigned to chemotherapy. Therefore, after three cycles of chemotherapy after gastrectomy, compliance is greatly reduced. Its long-term publication is still pending for mature results.

The FLOT4 study evaluated two regimens of perioperative chemotherapy, demonstrating a better median survival with the FLOT versus ECF/ECX regimens (50 versus 35 months), against high toxicity, with 14 more patients alive at 36 months; however, the ECF arm included 11 more N + patients. Data with longer follow-up from this study are still missing to determine if it is appropriate to abandon the ECF regimen altogether [13].

Regarding adjuvant chemotherapy, the CLASSIC trial randomised 1,035 patients to D2 gastrectomy with or without 6 months of adjuvant chemotherapy. After a median follow-up of 62.4 months, it reported beneficial results in overall survival in the adjuvant-treatment arm; however, only 66.5% of patients were able to complete treatment, against grades 3–4 toxicity in 56% of them, resulting highly toxic [14].

At the same time, Kang *et al* [16] published the ARTIST study [15], with an update in 2015 after 7 years of follow-up. In the latter trial, 458 patients with gastric cancer were randomised to chemotherapy (six cycles of capecitabine) or radiochemotherapy (two cycles of chemotherapy with capecitabine plus radiotherapy – 45 Gy in 25 sessions – plus two cycles of chemotherapy) after D2 resection. After a minimum follow-up of 5 years, 77 patients (33.8%) in the chemotherapy arm and 60 (26.1%) in the radiochemotherapy arm experienced recurrence. Locoregional failure was 9.6% (29 patients in the chemotherapy and 15 in the radiochemotherapy arms; *p* = 0.03); in patients with compromised nodes, radiochemotherapy significantly decreased locoregional recurrence compared to chemotherapy (14.5% versus 6.4%, *p* = 0.009). After 7 years of follow-up, subgroup analysis confirms again that radiochemotherapy significantly improves disease-free survival in patients with lymph node involvement and with intestinal histotype [16].

In 2018, the CRITICS study [17] was published, in which 788 patients with stage IB-IVA resectable gastric or gastroesophageal adenocarcinoma were enrolled. Patients were randomised to neoadjuvant and adjuvant chemotherapy (*n* = 393) or neoadjuvant chemotherapy followed by postoperative radiochemotherapy (*n* = 395). After surgery, 233/393 patients (59%) started chemotherapy and 245/395 (62%) started radiochemotherapy. After a median follow-up of 61.4 months, there was no statistically significant difference regarding overall survival (*p* = 0.90). During postoperative treatment, chemotherapy resulted in higher toxicity and lower treatment compliance than radiochemotherapy. Grades 3–4 acute toxicity was 48% and 9% in the chemotherapy group, respectively, and 41% and 4% in the radiochemotherapy group, respectively. The worst toxicity was neutropenia, which occurred more frequently in the chemotherapy arm (34%) versus the radiotherapy arm (4%).

Recently, the interim results of ARTIST 2 have been published. This early report concluded that in patients with curatively D2-resected, stage II/III, node-positive GC, adjuvant SOX (S-1 plus oxaliplatin) or SOXRT (S-1 plus oxaliplatin plus chemoradiotherapy) was effective in prolonging DFS, when compared to S-1 monotherapy [29]. When analysing the toxicities of each scheme, peripheral neuropathy, which may be irreversible, occurs in a greater percentage with the SOX scheme than that with the scheme that includes radiochemotherapy; therefore, it continues to be the standard for our institution.

Regarding publications of clinical-practice results of treatments with adjuvant or neoadjuvant therapies in Chile, the published experience comes exclusively from experiences with adjuvant radiochemotherapy from different reference cancer centres (PUC-INC-IRAM-FALP), with global 5-year survival rates varying between 37.5% and 54% [20–24], similar to those reported in randomised studies.

To date, there are no published data on local clinical practice results with chemotherapy alone, either as an adjuvant or as a perioperative treatment.

The **efficacy** of adjuvant therapy with radiochemotherapy and/or perioperative chemotherapy is well established with level-1 evidence; however, this should not be confused with the **effectiveness** of the therapy. Efficacy is understood as the benefit achieved in clinical studies with well-defined, selected patients; however, effectiveness is the benefit in the real world, outside of clinical studies.

In gastric cancer treatment, as in other tumours, there are data available that allow us to conclude that only radiochemotherapy has shown to have both efficacy and effectiveness, in contrast to perioperative chemotherapy, which has only been shown to have efficacy, although its effectiveness is unknown [25–26].

Furthermore, in local clinical practice, if only adjuvant or perioperative chemotherapy is performed, it should be clear that it is necessary to perform a microsatellite instability (MSI) study since chemotherapy worsens prognosis in cases with MSI [27, 28].

Taking into account the global increase in the incidence of this disease and the recently-described evidence, it is essential to know the oncologic results with other regimens in daily practice and, thus, to be able to estimate the impact of adjuvant treatments in our country.

Our retrospective series is the largest one published in our country, with results similar to those described in different international randomised and retrospective studies, which supports the use of adjuvant radiochemotherapy in our institution and country.

## Conclusion

In operated patients with locoregionally advanced gastric cancer treated with adjuvant radiochemotherapy with curative intent, 5-year overall survival reported from local clinical practice is similar to that reported in randomised series and supports its use as an effective treatment for this type of patients in our country.

## Funding statement

We did not receive any financial support.

## Conflicts of interest

The authors declare no conflict of interest.

## Figures and Tables

**Table 1.** Distribution between T, N and stage according to AJCC 8th ed. [4]

	pN0	pN1	pN2	pN3a	pN3b	Total (%)
pT1	0	8	3	1	0	12 (5.6)
pT2	2	11	8	2	1	24 (11.2)
pT3	14	6	13	6	5	44 (20.5)
pT4a	16	19	43	37	19	134 (62.6)
Total (%)	32 (14.9)	44 (20.5)	67 (31.3)	46 (21.5)	25 (11.6)	214 (100)

Stage: II-A

II-B

III

**Table 2.** Patient, treatment and tumour-related characteristics.

	Nº patients (214)	% (100)
Age Median Range	62 (23–85)	
Age <65 years ≥65 years	116 98	54% 46%
Sex Male Female	154 60	72% 28%
Type of surgery Subtotal gastrectomy Total gastrectomy	84 130	39.2% 61.8%
Type of dissection D1 D2	47 167	21.9% 78%
N examined nodes	Mean = 21.6 nodes. <16 nodes (84 patients) >16 nodes (130 patients) <10 nodes (29 patients)	
Resection edge R0 R1 R2	203 10 1	93.9% 4.6% 0.5%
Chemotherapy schedule 5 FU Capecitabine Cisplatin No chemotherapy	67 129 3 15	31.3% 60.2% 1.4% 7%
Type of radiotherapy WAI* Bed + nodes	69 145	32.2% 67.8%
Signet-ring cells Yes No	44 170	20.5% 79.5%
Time from surgery to RT/CT start <8 weeks >8 weeks	102 112	47.6%. 52.4%

* WAI: total abdomen irradiation.

RC/CT: radiochemotherapy

**Table 3.** Multivariate analysis of factors influencing OS for all patients.

	HR	*p*-value
N nodes involved	1.40	0.00
Age (≥65 years)	1.78	0.00
Time from surgery to RT/CT start	1.07	0.68
Chemotherapy	1.09	0.12
R edges	1.10	0.76
Signet-ring cells	0.96	0.68
Tumour stage	1.35	0.01
Type of nodal dissection	1.10	0.63
Type of surgery	1.07	0.65
Type of radiotherapy	0.81	0.24
Sex	1.41	0.08
